# Supplementary material for: Mapping the sugar dependency for rational generation of a DNA-RNA hybrid-guided Cas9 endonuclease
Source: Nat Commun. 2017 Nov 20;8:1610. doi: 10.1038/s41467-017-01732-9 (PMC5694763; doi:10.1038/s41467-017-01732-9)
Supplement: Supplementary file 1 — Supplementary Information [file 41467_2017_1732_MOESM1_ESM.pdf]

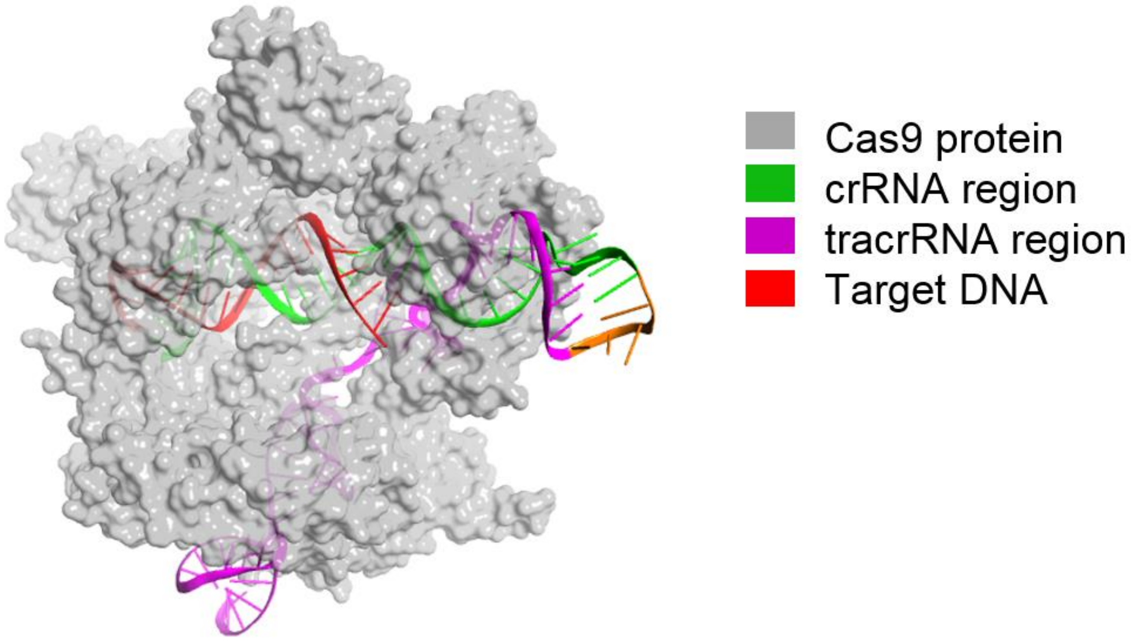

Supplementary Figure 1. Structure of the Cas9:sgRNA:targetDNA complex. Cas9 protein surface is depicted in grey, the equivalent crRNA region of the sgRNA in green, and the equivalent tracrRNA region in the sgRNA in purple. Target DNA is red. Structure derived from PDB 4OO8 [2].

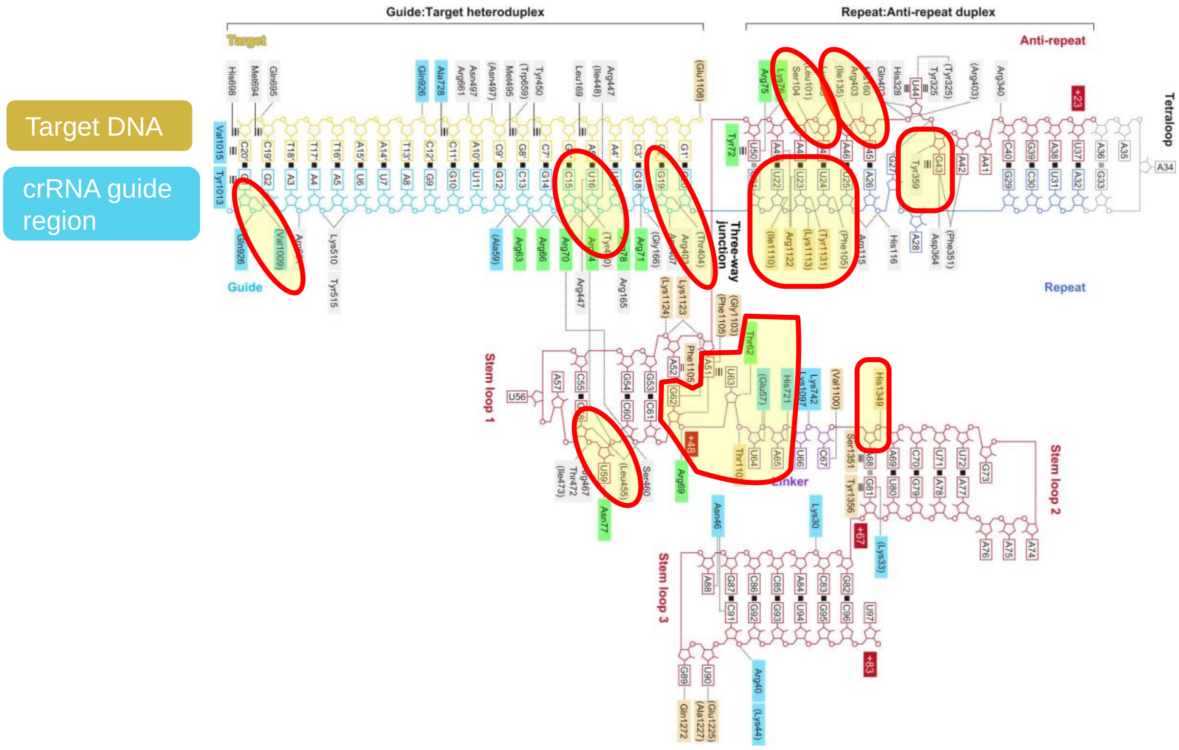

Supplementary Figure 2. Identified hydrogen bonds involving the 2'hydroxyl groups across the sgRNA molecule. All resides where the hydrogen bonds involving the 2' hydroxyl group which were considered for designs are highlighted in. Figure modified from work by Nishimasu and colleagues [2], with permission from Elsevier.

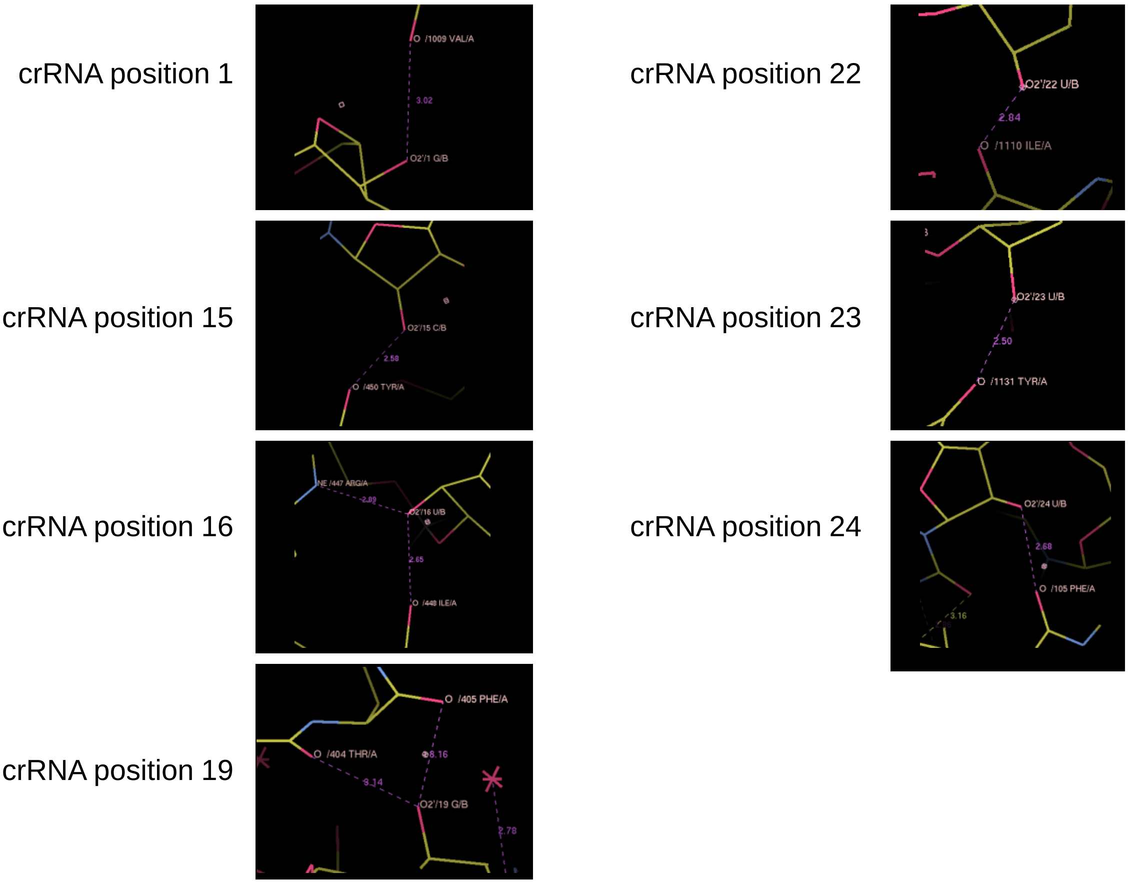

Supplementary Figure 3. Identified hydrogen bonds at the crRNA 2'hydroxyl group. RNA and protein are depicted as sticks with the residues involved marked and the identified hydrogen bond highlighted in purple with associated distance. Images derived from PDB structure 4OO8 [2].

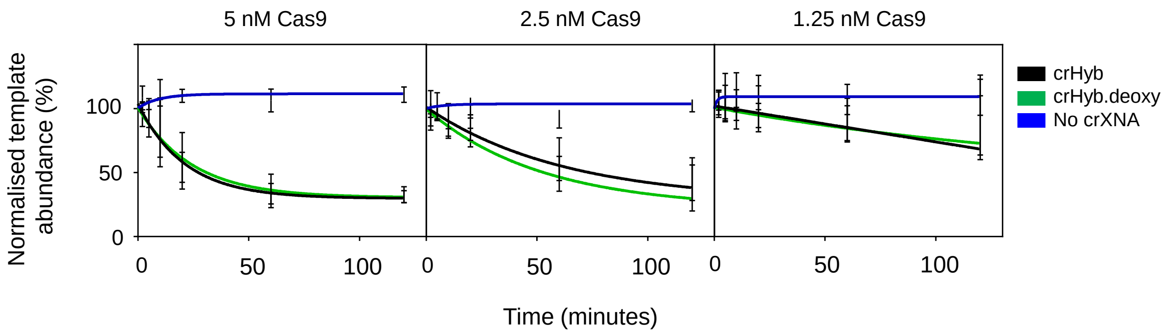


```
                |------ guide ------|---- cr repeat -----|
crHyb:          gCCAGTAGCCAGCCccGTcCGuuuTAGAGCTATGCTGTTTTG
crHyb.deoxy:    gCCAGUAGCCAGCCccGUcCGuuuUAGAGCUAUGCUGUUUUG
                |   |    |    |    |    |    |    |    |
                1   5    10   15   20   25   30   35   40

rna  DNA deoxy Uracil
```

Supplementary Figure 4. Methyl group within thymine residues do not affect cleavage activity. Cleavage efficiency of Cas9:tracrRNA complexed with crHyb (black), crHyb.deoxy (green) or no crXNA (blue) were assessed by *in vitro* biochemical cleavage and qPCR detection of template abundance at 5 nM (left), 2.5 nM (middle) or 1.25 nM (right) Cas9 protein concentration at multiple time points. Plots show mean ± standard deviation, n=4. crXNA sequence are shown in the lower panel, with DNA nucleotides in uppercase black and RNA nucleotides in lowercase red and deoxy Uracil residues in underlined green.

A **B**

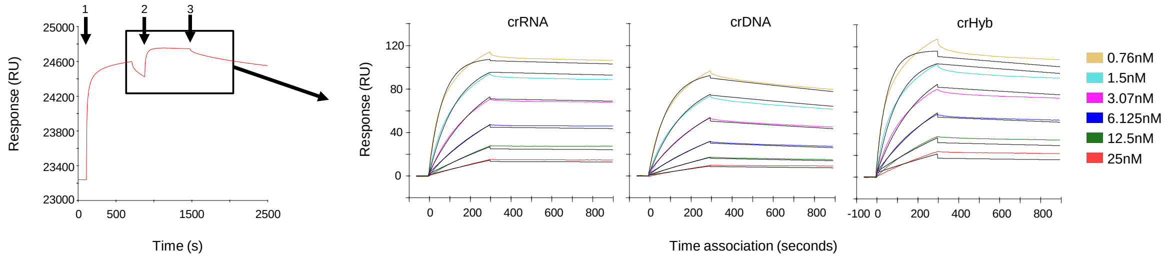

C

	Cas9-tracrRNA complex	
	Kd (pM)	t½ (mins)
RNA	81	240
DNA	584	42
crHyb	195	78

D

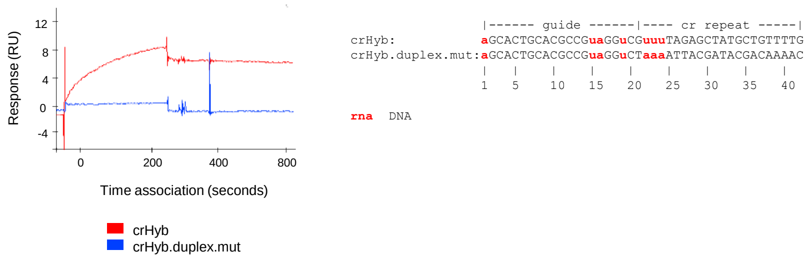

Supplementary Figure 5. Biophysical characterisation of Cas9:tracrRNA:cr interactions. (A) dCas9 was immobilised using standard amine coupling on a Biacore chip. Injection protocol: (arrow 1) Saturation of dCas9 with 100nM tracrRNA, (arrow 2) injection of crXNA molecules to measure binding affinity and (arrow 3) wash-out step to measure dissociation. **(B)** Sensograms showing binding of crXNA molecules to dCas9 saturated with tracrRNA; cXNA concentration range up to 200nM. **(C)** Binding constants and dissociation half-lives of Cas9:tracrRNA:cr complexes. **(D)** Binding of 100nM crHyb (red) or 100nM crHyb.duplex.mut (blue) to saturated Cas9:tracrRNA complexes, with corresponding sequence shown to the right, with DNA nucleotides in uppercase black and RNA nucleotides in lowercase red.

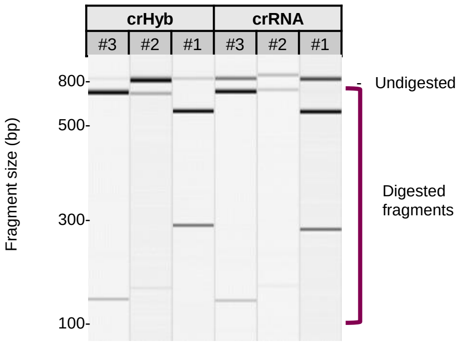


```
            |------ guide ------|---- cr repeat -----|
#1 crRNA:  agcacugcacgccguaggucguuuuagagcuaugcuguuuug
#1 crDNA:  AGCACTGCACGCCGTAGGTCGTTTTAGAGCTATGCTGTTTTG
#1 crHyb:  aGCACTGCACGCCGuaGGuCGuuuTAGAGCTATGCTGTTTTG

#2 crRNA:  caaguucagcguguccggcgguuuuagagcuaugcuguuuug
#2 crDNA:  CAAGTTCAGCGTGTCCGGCGGTTTTAGAGCTATGCTGTTTTG
#2 crHyb:  cAAGTTCAGCGTGTccGGcGguuuTAGAGCTATGCTGTTTTG

#3 crRNA:  gagcuggacggcgacguaaaguuuuagagcuaugcuguuuug
#3 crDNA:  GAGCTGGACGGCGACGTAAAGTTTTAGAGCTATGCTGTTTTG
#3 crHyb:  gAGCTGGACGGCGAcgTAaAguuutagagctatgctgttttg
            |   |    |    |    |    |    |    |    |
            1   5    10   15   20   25   30   35   40

rna  DNA
```

Supplementary Figure 6. Electropherogram of QIAxcel pseudogel image showing fragment sizes obtained from target DNA digestion with Cas9:tracrRNA and crHyb or crRNA complexes with three guide sequence to different regions in the target DNA sequence. crXNA sequences are shown on the right panel with DNA nucleotides in uppercase black and RNA nucleotides in lowercase red.

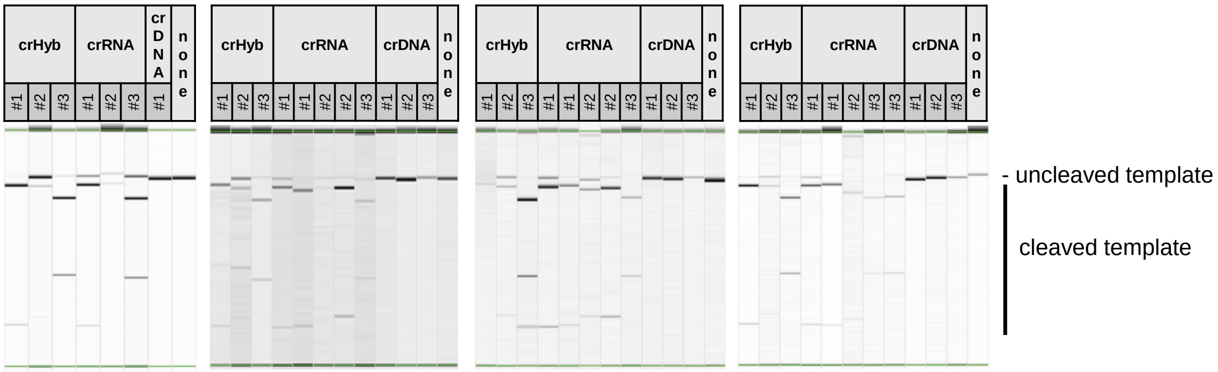

Supplementary Figure 7. Exemplar raw data used for Figure 1c. Electropherogram of QIAxcel pseudogel image showing fragment sizes obtained from target DNA digestion with Cas9:tracrRNA and crRNA, crHyb or crDNA complexes with three guide sequence to different regions in the target DNA sequence.

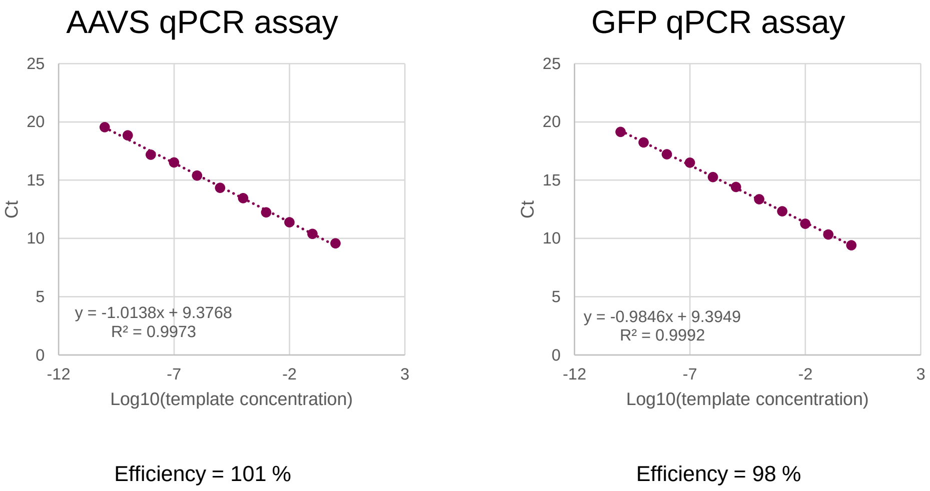

Supplementary Figure 8. Template depletion qPCR probe validation. Assessment of amplification linearity of qPCR probe assay for determining relative concentration of AAVS1 and GFP templates. Probes were tested on limiting dilutions of purified templates with amplification efficiencies shown.

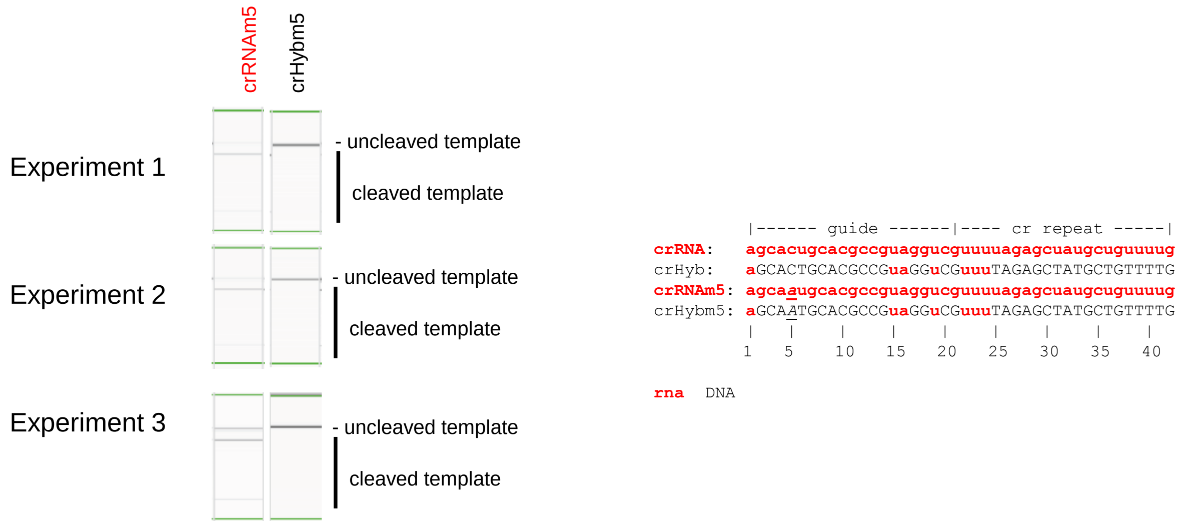

Supplementary Figure 9. Exemplar raw data used for Figure 2b. Electropherogram of QIAxcel pseudogel image showing fragment sizes obtained from target DNA digestion with Cas9:tracrRNA complexed with mismatched crRNA and crHyb. crXNA sequences are shown on the right panel with DNA nucleosides in uppercase black and RNA nucleosides in lowercase red.

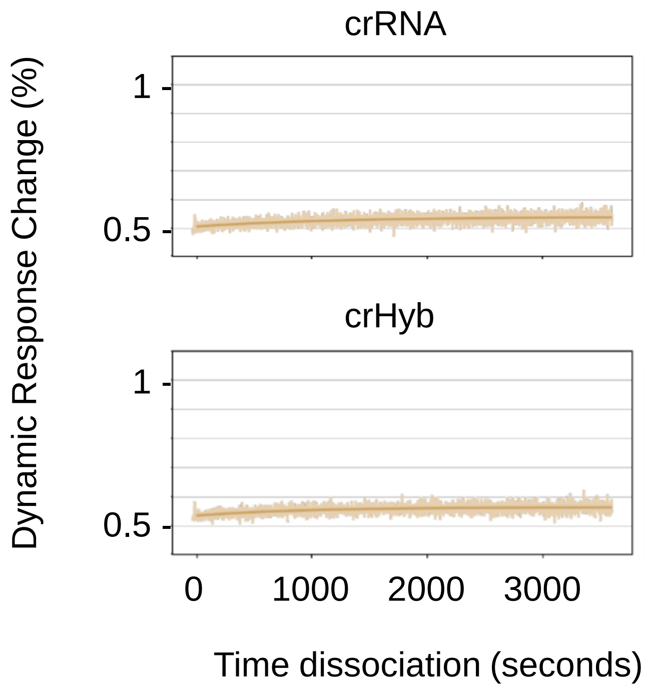

Supplementary Figure 10. Dissociation curves of target bound Cas9:tracrRNA:crRNA or Cas9:tracrRNA: crHyb complexes.

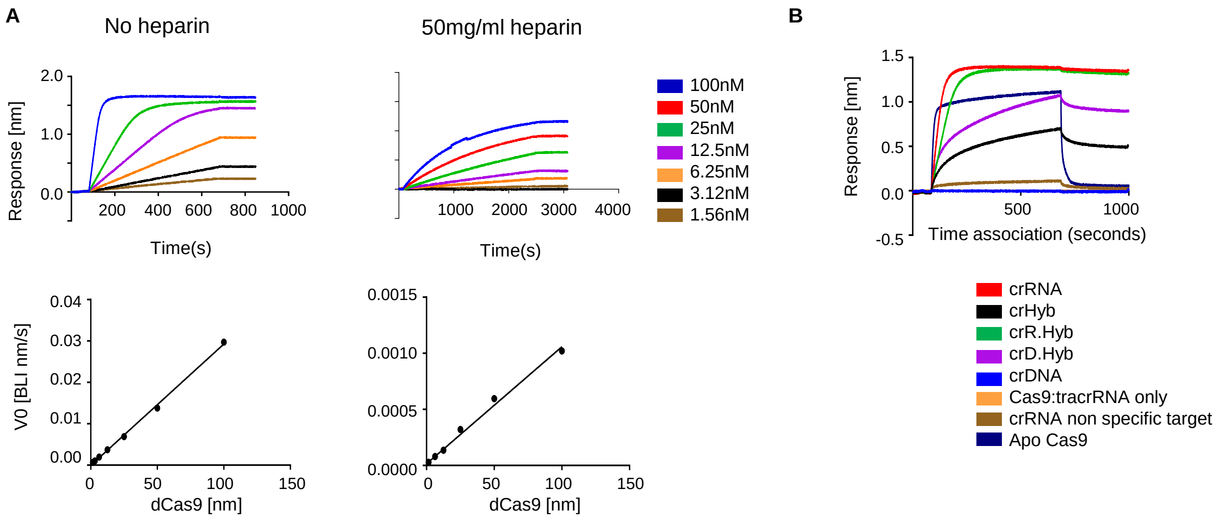

Slope	29.32e-005 ± 5.48E-6
Y-intercept	-7.016e-005 ± 0.0002392

Slope	1.043e-005 ± 5.044E-7
Y-intercept	1.471e-005 ± 2.202E-5

Supplementary Figure 11. Biophysical characterisation of Cas9:tracrRNA:crXNA interactions. (A) BLI binding kinetics (upper) of Cas9:tracrRNA:crRNA binding to immobilised AAVS1 target at multiple concentrations in buffer without (left) or with (right) 50mg ml^{-1} Heparin. Lower panel shows the calculated velocity constant and table shows the results of linear regression. **(B)** BLI sensogram showing association kinetics in heparin free buffer to immobilised AAVS1 target DNA of Cas9:tracrRNA complexed with crRNA (red), crHyb (black), crDNA (blue), crR.Hyb (green) or crD.Hyb (purple), Cas9:tracrRNA alone (orange), or Apo Cas9 (dark blue) and Cas9:tracrRNA:crRNA binding to a non-specific target (brown).

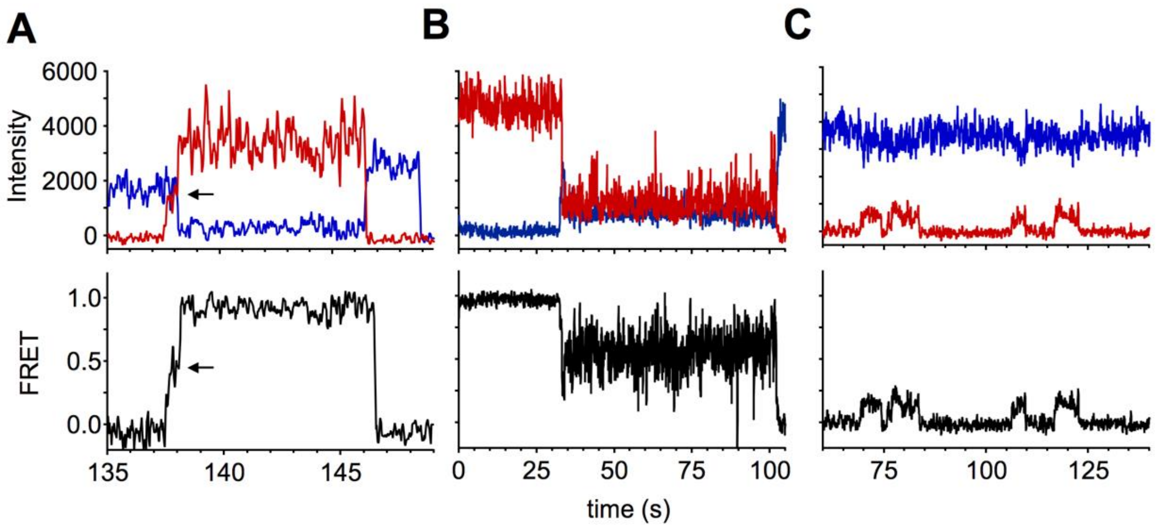

Supplementary Figure 12. Single-molecule fluorescence intensity and calculated FRET trajectories. Representative single-molecule trajectories displaying fluorophore intensities (top) of Cy3 donor (blue) and Cy5 acceptor (red) with calculated FRET trajectories (bottom). **(A)** During dCas9-crRNA complex binding and stable heteroduplex formation an anti-correlated increase in acceptor and decrease in donor intensities is observed resulting in a high FRET state (left). Transient mid-FRET (0.5) intermediate is indicated by an arrow. **(B)** dCas9-crRNA complex undergoing irreversible transition from stable heteroduplex state to dynamic state (middle). **(C)** A minor population of traces show transient low FRET state. Traces are 5 point averaged.

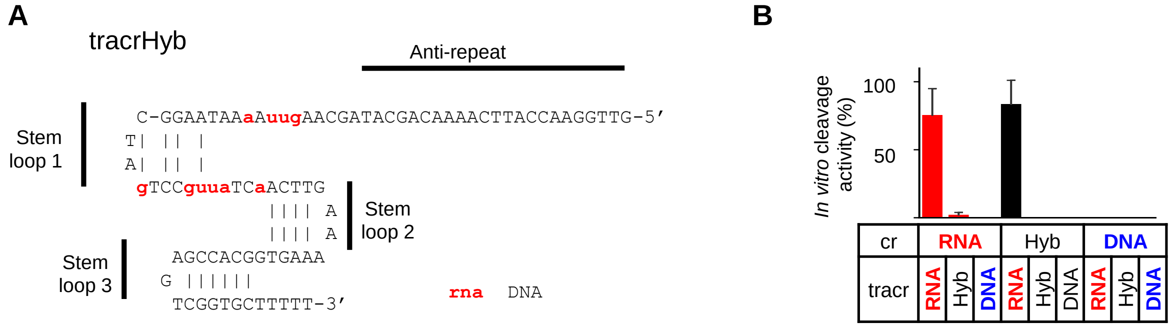

Supplementary Figure 13. Biochemical and cellular characterisation of tracrHyb. (A) Design of single tracrHyb, bases coloured as in Figure 1a. **(B)** Cleavage activity of Cas9 in complex with crXNA and tracrXNA molecules composed of RNA, DNA or Hybrid bases.

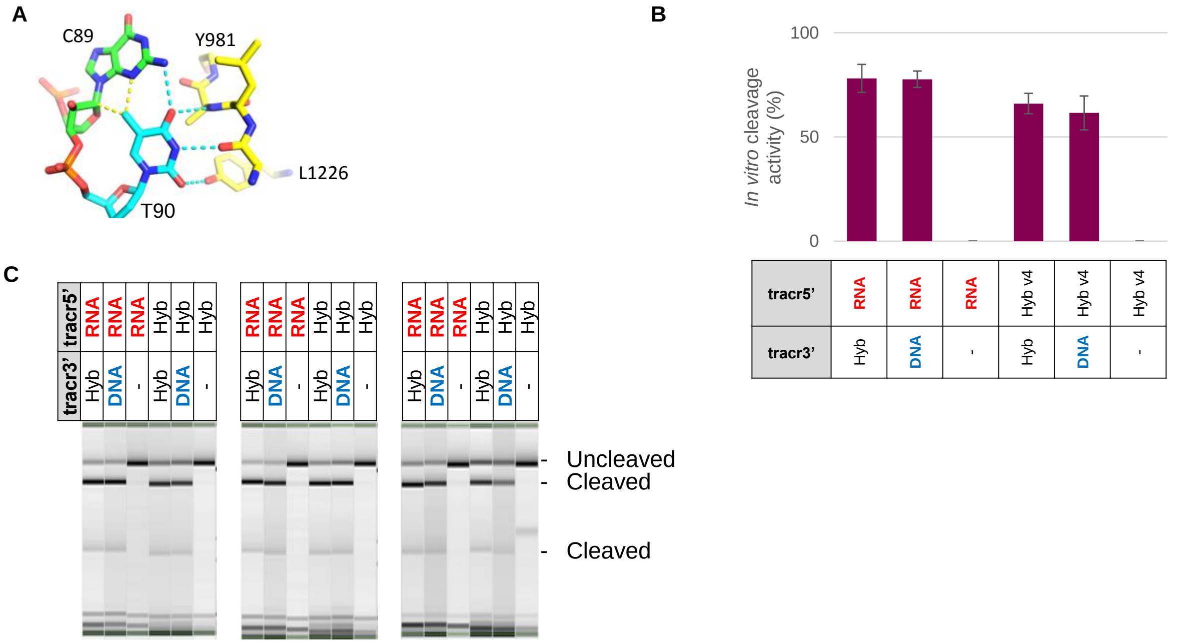

Supplementary Figure 14. Effect of DNA in the tracr3' region. (A) Protein is depicted as sticks with yellow carbon atoms. The base change U90T (coloured cyan) has been modelled using PDB coordinates 4OO8. Hydrogen bonds have been depicted as cyan dotted lines. Clashes between the methyl of the thymine and C89 are shown as yellow dotted lines. T90 makes hydrogen bonds with Y981 side chain and L1226 backbone. **(B)** Cleavage activity of a Hybrid or fully DNA tracr3' molecule was assessed when complexed with Cas9:crHyb:tracr5' RNA or Cas9: crHyb:tracr5' Hyb v4. **(C)** Electropherogram of QIAxcel pseudogel image of data used to generate in **(B)**.

		crXNA	RNA	Hyb	RNA	Hyb	RNA	Hyb	RNA	Hyb	RNA	Hyb	RNA	Hyb	RNA	Hyb	RNA	Hyb	RNA	Hyb	RNA	Hyb	RNA	Hyb	RNA	Hyb	RNA	Hyb	RNA	Hyb	RNA	Hyb	RNA
		tracrXNA 5'	tracrRNA	tracrRNA	RNA	RNA	Hyb v1	Hyb v1	RNA	RNA	Hyb v1	Hyb v1	RNA	RNA	Hyb v2	Hyb v2	Hyb v3	Hyb v3	Hyb v4	Hyb v4	Hyb v5	Hyb v5	Hyb v6	Hyb v6	Hyb v7	Hyb v7	Hyb v8	Hyb v8	Hyb v9	Hyb v9	Hyb v10	Hyb v10	tracrHyb.v2
crXNA	tracrXNA 5'	tracrXNA 3'			-	-	-	-	RNA	RNA	RNA	RNA	Hyb	Hyb	Hyb	Hyb	Hyb	Hyb	Hyb	Hyb	Hyb	Hyb	Hyb	Hyb	Hyb	Hyb	Hyb	Hyb	Hyb	Hyb	Hyb	Hyb	-
Hyb	tracrRNA		0.9999
RNA	RNA	-	0.9999	0.9999
Hyb	RNA	-	0.0001	0.0001	0.0001
RNA	Hyb v1	-	0.0001	0.0001	0.0001	0.9999
Hyb	Hyb v1	-	0.0001	0.0001	0.0001	0.9999	0.9999
RNA	RNA	RNA	0.9071	0.9915	0.9999	0.0001	0.0001	0.0001
Hyb	RNA	RNA	0.9983	0.9999	0.9999	0.0001	0.0001	0.0001	0.9999
RNA	Hyb v1	RNA	0.0001	0.0001	0.0001	0.9999	0.9999	0.9999	0.0001	0.0001
Hyb	Hyb v1	RNA	0.0001	0.0001	0.0001	0.9999	0.9999	0.9999	0.0001	0.0001	0.9999
RNA	RNA	Hyb	0.9999	0.9999	0.9999	0.0001	0.0001	0.0001	0.9999	0.9999	0.0001	0.0001
Hyb	RNA	Hyb	0.0126	0.0478	0.2237	0.0001	0.0001	0.0001	0.9717	0.6969	0.0001	0.0001	0.2471
RNA	Hyb v2	Hyb	0.0001	0.0001	0.0001	0.9561	0.8758	0.8758	0.0001	0.0001	0.9997	0.8758	0.0001	0.0001
Hyb	Hyb v2	Hyb	0.0001	0.0001	0.0001	0.9999	0.9999	0.9999	0.0001	0.0001	0.9999	0.9999	0.0001	0.0001	0.8758
RNA	Hyb v3	Hyb	0.0077	0.0311	0.1613	0.0001	0.0001	0.0001	0.9399	0.5921	0.0001	0.0001	0.1798	0.9999	0.0001	0.0001
Hyb	Hyb v3	Hyb	0.0001	0.0001	0.0013	0.0001	0.0001	0.0001	0.0967	0.0154	0.0001	0.0001	0.0015	0.9991	0.0001	0.0001	0.9998
RNA	Hyb v4	Hyb	0.0001	0.0002	0.0021	0.0001	0.0001	0.0001	0.1372	0.0239	0.0001	0.0001	0.0025	0.9998	0.0001	0.0001	0.9999	0.9999
Hyb	Hyb v4	Hyb	0.0001	0.0001	0.0001	0.0001	0.0001	0.0001	0.0049	0.0005	0.0001	0.0001	0.0001	0.7635	0.0021	0.0001	0.8471	0.9999	0.9999
RNA	Hyb v5	Hyb	0.5955	0.8689	0.9956	0.0001	0.0001	0.0001	0.9999	0.9999	0.0001	0.0001	0.997	0.9996	0.0001	0.0001	0.9982	0.3155	0.4044	0.0271
Hyb	Hyb v5	Hyb	0.0001	0.0001	0.0001	0.0186	0.0085	0.0085	0.0001	0.0001	0.1167	0.0085	0.0001	0.0003	0.9639	0.0085	0.0006	0.0993	0.0686	0.6342	0.0001
RNA	Hyb v6	Hyb	0.0001	0.0001	0.0001	0.5167	0.3507	0.3507	0.0001	0.0001	0.9116	0.3507	0.0001	0.0001	0.9999	0.3507	0.0001	0.0011	0.0007	0.0305	0.0001	0.9999
Hyb	Hyb v6	Hyb	0.0001	0.0001	0.0001	0.9701	0.9056	0.9056	0.0001	0.0001	0.9999	0.9056	0.0001	0.0001	0.9999	0.9056	0.0001	0.0001	0.0001	0.0016	0.0001	0.948	0.9999
RNA	Hyb v7	Hyb	0.0001	0.0001	0.0002	0.0001	0.0001	0.0001	0.0266	0.0033	0.0001	0.0001	0.0003	0.9699	0.0003	0.0001	0.9878	0.9999	0.9999	0.9999	0.1154	0.2817	0.0057	0.0002
Hyb	Hyb v7	Hyb	0.0001	0.0001	0.0001	0.9999	0.9999	0.9999	0.0001	0.0001	0.9999	0.9999	0.0001	0.0001	0.9518	0.9999	0.0001	0.0001	0.0001	0.0001	0.0001	0.0175	0.5032	0.9669	0.0001
RNA	Hyb v8	Hyb	0.0001	0.0007	0.006	0.0001	0.0001	0.0001	0.266	0.0575	0.0001	0.0001	0.0071	0.9999	0.0001	0.0001	0.9999	0.9999	0.9999	0.9999	0.6181	0.029	0.0002	0.0001	0.9999	0.0001
Hyb	Hyb v8	Hyb	0.0012	0.0059	0.0413	0.0001	0.0001	0.0001	0.6747	0.2521	0.0001	0.0001	0.0474	0.9999	0.0001	0.0001	0.9999	0.9999	0.9999	0.9875	0.9425	0.004	0.0001	0.0001	0.9999	0.0001	0.9999
RNA	Hyb v9	Hyb	0.0001	0.0001	0.0001	0.3877	0.2454	0.2454	0.0001	0.0001	0.8282	0.2454	0.0001	0.0001	0.9999	0.2454	0.0001	0.0022	0.0013	0.0524	0.0001	0.9999	0.9999	0.9999	0.0106	0.3755	0.0004	0.0001
Hyb	Hyb v9	Hyb	0.0001	0.0001	0.0001	0.9999	0.9999	0.9999	0.0001	0.0001	0.9999	0.9999	0.0001	0.0001	0.9999	0.9999	0.0001	0.0001	0.0001	0.0001	0.0001	0.1376	0.9337	0.9999	0.0001	0.9999	0.0001	0.0001	0.8622
RNA	Hyb v10	Hyb	0.0001	0.0001	0.0001	0.0001	0.0001	0.0001	0.0001	0.0001	0.0013	0.0001	0.0001	0.0456	0.1741	0.0001	0.0688	0.8966	0.8311	0.9999	0.0002	0.9999	0.6727	0.1471	0.9911	0.0001	0.6393	0.2402	0.7934	0.0016
Hyb	Hyb v10	Hyb	0.0001	0.0001	0.0001	0.2465	0.1436	0.1436	0.0001	0.0001	0.6771	0.1436	0.0001	0.0001	0.9999	0.1436	0.0001	0.0051	0.0031	0.1002	0.0001	0.9999	0.9999	0.9999	0.0228	0.2371	0.0011	0.0001	0.9999	0.723	0.9093
RNA	tracrHyb.v2		0.9999	0.9999	0.9999	0.0001	0.0001	0.0001	0.9994	0.9999	0.0001	0.0001	0.9999	0.1091	0.0001	0.0001	0.0744	0.0004	0.0007	0.0001	0.966	0.0001	0.0001	0.0001	0.0001	0.0001	0.002	0.0161	0.0001	0.0001	0.0001	0.0001
Hyb	tracrHyb.v2		0.0001	0.0001	0.0001	0.0029	0.0012	0.0012	0.0001	0.0001	0.025	0.0012	0.0001	0.0027	0.722	0.0012	0.0045	0.3366	0.2568	0.9332	0.0001	0.9999	0.9925	0.6724	0.6563	0.0027	0.1315	0.0245	0.9984	0.0307	0.9999	0.9999	0.0001

Supplementary Figure 15. Significance test of cleavage efficiencies from Figure 6b. Multiple comparison statistics using two-way ANOVA analysis was conducted and resultant p-values are shown and statically significant results (p<0.05) highlighted gold.

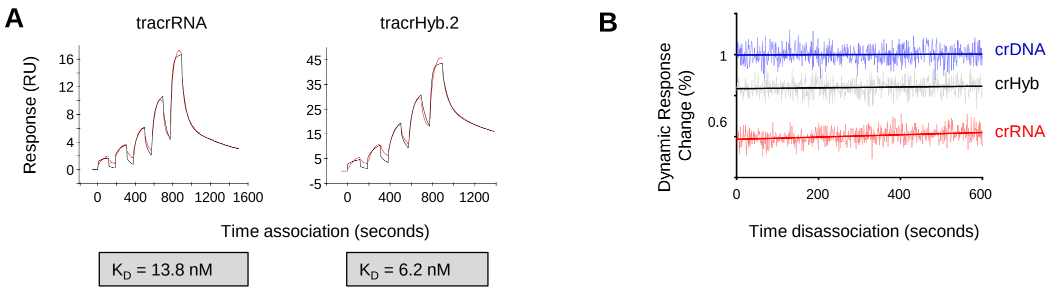

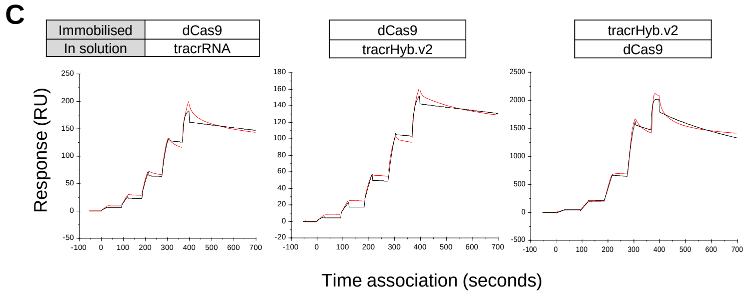

D

Immobilised	In solution	Kon [M-1 s-1]	koff [s-1]	Kd [M]
dCas9	tracrRNA	1.04E6 ± 2.6E4	3.20E-4 ± 1.3E-5	3.08E-10 ± 5E-12
dCas9	tracrHyb.v2	8.95E5 ± 4.3E4	2.75E-4 ± 4.0E-6	3.08E-10 ± 1.4E-11
tracrHyb.v2	dCas9	2.55E6 ± 3.3E5	1.29E-3 ± 5.5E-5	5.1E-10 ± 4.2E-11

Supplementary Figure 16. Biophysical characterisation of dCas9:tracrRNA and dCas9:tracrHyb.2 interactions. (A) Increasing concentrations (from 1.2nM to 100nM) of tracrRNA or tracrHyb.2 were injected and followed by dissociation delay in a single experimental cycle (Single Cycle Kinetics mode). Sensograms were fitted using 2-step binding model. **(B)** Dissociation curves from SwitchSENSE measurements of target engaged Cas9:tracrHyb.v2 complexed with crRNA (red), crHyb (black) or crDNA (blue). **(C)** Left panel shows tracrRNA binding to immobilised dCas9; 1.2 nM, 3.7 nM, 11.1 nM, 33 nM, 100 nM tracrRNA injected over dCas9 containing surface. Middle panel shows tracrHyb.2 binding to immobilised dCas9; 1.2 nM, 3.7 nM, 11.1 nM, 33 nM, 100 nM tracrHyb.v2 injected over dCas9 containing surface. Right panel shows binding of dCa9 to immobilised biotinylated tracrHyb.2 ; 1.2 nM, 3.7 nM, 11.1 nM, 33 nM, 100 nM dCas9 injected over tracrHyb.2 derivatised surface. **(D)** Kinetic parameters of tracrRNA binding determined by fitting of 1:1 single step model of binding (n=3, standard deviations reported).

tracrXNA	crXNA	Biochem activity (%)	SwitchSENSE relative activity (%)	BLI (%)	Cell (%)
**tracrRNA**	crRNA	90.4 ± 4.4	100 ± 8.1	100	58.7 ± 12.6
	crDNA	0 ± 0	5.83 ± 7.3	-2.2 ± 0.3	3.4 ± 1.7
	crHyb	87.7 ± 1.5	26.4 ± 1.2	3.6 ± 0.2	4.7 ± 2.5
	crR.Hyb	n.d.	n.d	48.8 ± 4.1	13.3 ± 7.7
	crD.Hyb	n.d.	75.2 ± 6.8	9.3 ± 2.7	4.2 ± 2.5
**tracrHyb.2**	crRNA	85.9 ± 4.1	13.2 ± 0.4	4.4 ± 0.7	17 ± 7.9
	crDNA	n.d.	0 ± 0	3.7 ± 0.2	3.6 ± 1.4
	crHyb	32.0 ± 6.9	0.76 ± 0.07	3.1 ± 0.5	3.6 ± 3.0
	crR.Hyb	n.d.	n.d.	2.8 ± 0.7	7.1 ± 3.8
	crD.Hyb	n.d.	n.d.	n.d.	3.1 ± 1.7

Supplementary Table 1. Summary of results. SwitchSENSE and BLI activities are expressed as association rates normalised to (crRNA + tracrRNA). Measurements were performed in triplicates or greater and standard deviations are reported.

	crRNA	crHyb	crD.Hyb	crR.Hyb
**tracrRNA**	18.3E+06	4.83E+06	8.44E+06	2.15E+06
**tracrHyb.2**	2.42 E+06	0.14E+06	n.d.	n.d.

Supplementary Table 2. Activity by SwitchSENSE. Association (on-) rate constants determined by SwitchSENSE [$M^{-1}s^{-1}$]. Dissociation in SwitchSENSE experiments was not observed, so it was assumed k_{off}=0; k_{on} = k_{obs}/[Cas9].

Oligo Name	Sequence
5′ Cy5-crRNA	5′Cy5-GCCAGUAGCCAGCCCCGUCC GUUUUAGAGCUAUGCUGUUUUG
5′ Cy5-cr.Hyb	5′Cy5-GCCAGTAGCCAGCCCCGTCC gUUUtagagctatgctgttttg
5′ Cy5-crD.Hyb	5′Cy5-GCCAGTAGCCAGCCCCGTCC GUUUUAGAGCUAUGCUGUUUUG
5′ Cy5-crR.Hyb	5′Cy5-GCCAGUAGCCAGCCCCGUCC GUUUUAGAGCTATGCTGTTTTG
5′ Cy5-crDNA	5′Cy5-GCCAGTAGCCAGCCCCGTCC gttttagagctatgctgttttg
5′ Bio-AAVS1-Cy3	5′Bio-ttttttGGTAG CGGTCTGAGC CAGTAGCCAG CCCCG TCCAG GGTGGTCACG AGGG-3′

Supplementary Table 3. Oligonucleotides used in the single molecule FRET assays. DNA nucleosides in black uppercase, RNA nucleosides in red lowercase. Cy3 donor is chemically linked to underlined T. Green: Target sequence. Orange: PAM site.
